# Epithelial-Mesenchymal Transition in Keratocystic Odontogenic Tumor: Possible Role in Locally Aggressive Behavior

**DOI:** 10.1155/2015/168089

**Published:** 2015-03-23

**Authors:** Wen-Qun Zhong, Gang Chen, Wei Zhang, Jian-Gang Ren, Zhong-Xing Wu, Yi Zhao, Bing Liu, Yi-Fang Zhao

**Affiliations:** ^1^State Key Laboratory Breeding Base of Basic Science of Stomatology (Hubei-MOST) and Key Laboratory of Oral Biomedicine Ministry of Education, School and Hospital of Stomatology, Wuhan University, 237 Luoyu Road, Wuhan 430079, China; ^2^Department of Oral and Maxillofacial Surgery, School and Hospital of Stomatology, Wuhan University, Wuhan 430079, China; ^3^Department of Prosthodontics, School & Hospital of Stomatology, Wuhan University, Wuhan 430079, China

## Abstract

The aim of this study is to clarify whether epithelial-mesenchymal transition (EMT) is involved in the pathogenesis and development of keratocystic odontogenic tumor (KCOT). The expression levels of EMT-related proteins and genes in normal oral mucosa (OM), radicular cyst (RC), and KCOT were determined and compared by real-time quantitative PCR and immunohistochemistry. Our data showed that the expression of epithelial markers E-cadherin and Pan-cytokeratin was significantly downregulated in KCOT with upregulation of mesenchymal markers N-cadherin compared to OM and RC. Importantly, TGF-*β*, a potent EMT inducer, and Slug, a master transcription factor, were also found highly expressed in KCOT. In addition, the results from Spearman rank correlation test and clustering analysis revealed the close relationship between Slug and MMP-9, which was further evidenced by double-labeling immunofluorescence that revealed a synchronous distribution for Slug with MMP-9 in KCOT samples. All the data suggested EMT might be involved in the locally aggressive behavior of KCOT.

## 1. Introduction

According to the new World Health Organization (WHO) classification in 2005, odontogenic keratocyst (OKC) has been recategorized as a benign odontogenic tumor under the name of keratocystic odontogenic tumor (KCOT), based on their aggressive biological behavior, tendency towards recurrence, and potential of malignant transformation [1]. The knowledge of KCOT has been ever increasing over the past few decades. However, the precise pathological process of KCOT remains ambiguous.

Recently, the epithelial-mesenchymal transition (EMT), first recognized as a feature of embryogenesis in the early 1980s, has been found to contribute to a wide range of physiological and pathological process. EMT is a process in which epithelial cells lose their polarity and cohesiveness as well as acquire mesenchymal morphology and increased motility, which is closely associated with the embryogenesis, fibrosis, and tumorigenesis [2, 3]. Previous studies including our previous job have revealed that EMT was involved in different stages of tumor progression, including invasion, acquisition of anoikis resistance, and metastasis [4, 5]. EMT can endow tumor cells with lethal and invasive behaviors, which are associated with poor clinical outcome and high recurrence rate in multiple tumor types [6, 7]. To our interest, the rests of Malassez, the origination of many epithelial odontogenic tumors including KCOT, also contain unique cell populations which are capable of undergoing EMT [8]. However, it is still unclear whether EMT is involved in the pathogenesis and development of KCOT or not.

In our present study, the expression levels of EMT-related genes and proteins in normal oral mucosa (OM), radicular cyst (RC), and KCOT samples were detected and compared by immunohistochemistry and real-time quantitative PCR. Meanwhile, the correlation between these tested EMT-related markers and their correlation with the well-known cell invasive markers (MMP-9) in KCOT were also demonstrated using the correlation analysis, followed by the hierarchical clustering analysis. To the best our knowledge, this is the first study to investigate EMT and its potential relationship with the aggressive behaviors in KCOT.

## 2. Materials and Methods

### 2.1. Specimens

Forty samples of KCOT, 20 samples of RC, and 10 samples of OM were collected at the Hospital of Stomatology, Wuhan University (Wuhan, China). Specimens were fixed in buffered 4% paraformaldehyde for 2-3 days and embedded in paraffin. The procedures were performed in the light of the national institutes of health guidelines regarding the use of human tissues. This study was approved by the review board of the Ethics Committee of Hospital of Stomatology, Wuhan University. Meanwhile, the basically clinical date is described in the Supplementary Materials (see Supplementary Material available online at http://dx.doi.org/10.1155/2015/168089).

### 2.2. Real-Time Quantitative PCR

Ten fresh samples of KCOT, 9 fresh samples of RC, and 7 fresh samples of OM were collected. Then, the epithelia of KCOT, RC, and OM samples were carefully dissected by using laser-capture microdissection (LCM) as our previous description [9]. Total RNA was isolated for the synthesis of cDNA, and then real-time quantitative PCR (qPCR) was carried out according to our previous study [4]. 18s rRNA was chosen as an internal control. The primer sequences designed for qPCR were presented in Supplementary Materials.

### 2.3. Immunohistochemistry

The immunohistochemistry was performed in accordance with our previous report [10]. Briefly, 4 *μ*m tissue sections were dewaxed in xylene, antigen retrieved by high pressure, and then incubated in 3% hydrogen peroxide at 37°C for 15 min. After being washed in phosphate-buffered saline (PBS) three times and blocked with goat serum, the sections were incubated with primary antibodies Pan-cytokeratin (Zsbio), E-cadherin (Cell Signaling Technology), N-cadherin (OriGene), Slug (Cell Signaling Technology), TGF-*β*1 (Proteintech), and MMP-9 (Santa Cruz) overnight at 4°C. Then, the antibody binding was detected by horseradish peroxidase-conjugated secondary antibody. Peroxidase activity was visualized by immersing the tissue sections in diaminobenzidine and haematoxylin in sequence.

### 2.4. Evaluation of Immunohistochemical Staining

For evaluation of immunostaining scores, five representative high-power fields were randomly selected at a magnification of 200 with light microscope (Leica) and counted by two blind researchers. The scores were all calculated as the summation of staining intensity (0, no staining; 1, mild staining; 2, moderate staining; 3, intense staining) and the percentage of positive cells (0, <10%; 1, 10%–25%; 2, 25%–50%; 3, 50–75%; 4, 75–100% of the KCOT and RC epithelial cells).

### 2.5. Double-Labeling Immunofluorescence

The expression of Slug and MMP-9 was detected by double-labeling immunofluorescence in the clinical samples of OM, RC, and KCOT, respectively. The method of double-labeling immunofluorescence was performed as our previous description [10].

### 2.6. Hierarchical Clustering and Statistical Analysis

In line with our previous description [9, 11], the immunostaining scores of each marker were converted into scaled values centered on zero. The hierarchical clustering analysis was conducted by using Cluster 3.0 and the results were intuitively visualized by using the Java TreeView 1.0.5.

Statistical analysis was performed by using GraphPad Prism 5.03 (GraphPad Software, Inc., La Jolla, CA) statistical packages. Student's *t*-test and Spearman rank correlation were conducted to analyze the differences in immunostaining scores among each group. *P* < 0.05 was considered significantly different.

## 3. Result

### 3.1. Significant Changes of EMT-Related Genes in KCOT

**Figure 1:**
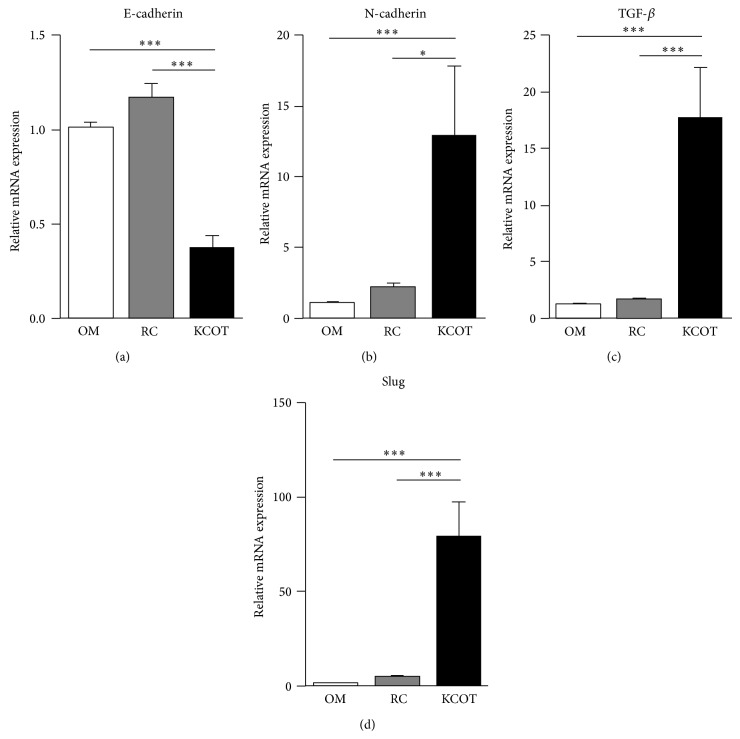
The mRNA expression levels of E-cadherin (a), N-cadherin (b), TGF-*β* (c), and Slug (d) in KCOT, RC, and OM tissue samples were detected by real-time qPCR analysis. Data are expressed as means accompanied by SEM. ^*^ *P* < 0.05 and ^***^ *P* < 0.001.

To evaluate the activation status of EMT in KCOT, the expression levels of EMT-related genes were detected in the samples of KCOT (*n* = 10), RC (*n* = 9), and OM (*n* = 7) by qPCR. Our results showed that the mRNA expression levels of the epithelial marker, E-cadherin, was significantly downregulated, while mesenchymal cell marker, N-cadherin, was significantly upregulated in KCOT compared to those in RC and OM (Figure 1). Meanwhile, the mRNA expression levels TGF-*β* and Slug were also higher in KCOT samples when compared to those in RC and OM samples. These results indicated an activation status of EMT in the development of KCOT.

### 3.2. Significant Changes of EMT-Related Proteins in KCOT

**Figure 2:**
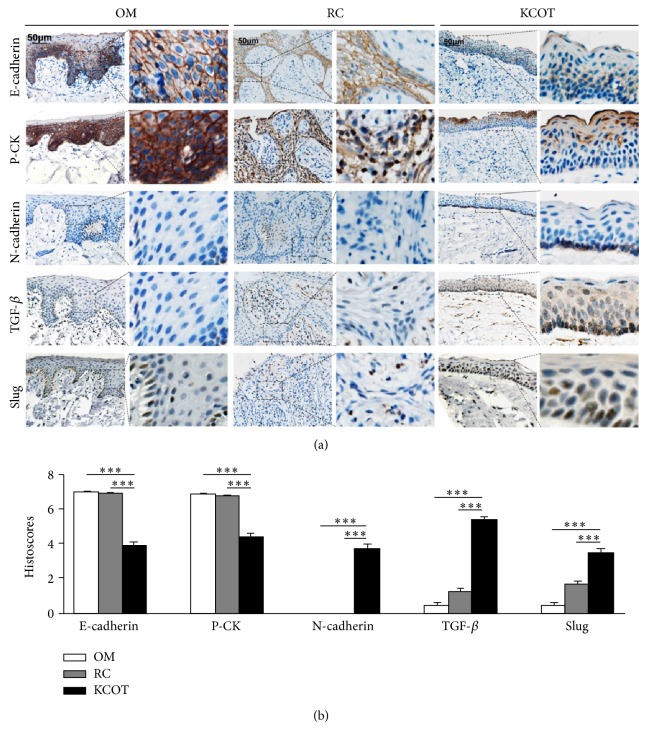
(a) The immunohistochemical staining of E-cadherin, P-CK, N-cadherin, TGF-*β*, and Slug in KCOT, RC, and OM tissue samples. (b) Immunostaining scores for the tested EMT-related markers in KCOT, RC, and OM samples. Data are presented as means accompanied by SEM. ^***^ *P* < 0.001.

To further explore the activation status of EMT in KCOT, immunohistochemistry was performed to detect the expression of EMT-related proteins in KCOT, RC, and OM samples, respectively. In detail, we found that the expression of E-cadherin and Pan-cytokeratin (P-CK) was restricted to the epithelial component in all investigated cases. The P-CK and E-cadherin were expressed throughout the epithelium including the basal and super layer cells in RC and OM. In sharp contrast, immunoreactivity of P-CK and E-cadherin in KCOT samples showed significant loss, especially in the basal layer cells (Figure 2(a)). Student's *t*-test statistical analysis showed that the expression levels of E-cadherin and P-CK in the epithelium of KCOT were significantly downregulated compared to those in RC and OM samples (*P* < 0.0001) (Figure 2(b)). In addition, the mesenchymal cell marker N-cadherin was negatively expressed in the epithelium of all the RC and OM samples. However, the expression of N-cadherin was positive in the epithelium of KCOT, especially in the basal layer cells (Figure 1(a)). Meanwhile, both EMT-inducing cytokines (TGF-*β*) and transcription factors (Slug) were also upregulated in KCOT samples when compared with RC and OM samples (Figure 1).

**Figure 3:**
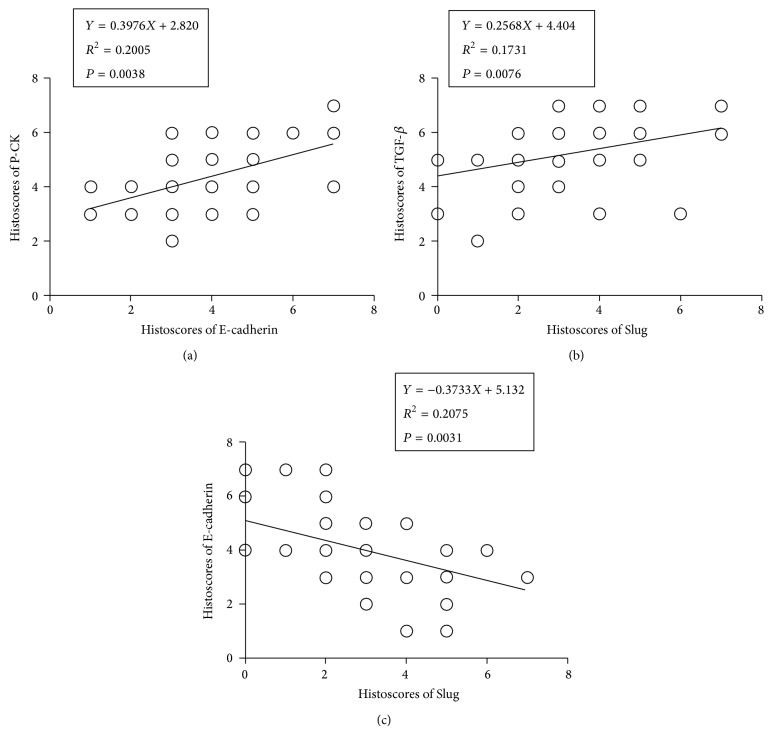
Spearman rank test analyses for immunostaining of some EMT-related markers in KCOT samples. (a) The expression level of E-cadherin indicated a significantly positive correlation with P-CK. (b) The expression level of Slug showed a significantly positive correlation with TGF-*β*. (c) The expression level of Slug displayed a significantly negative correlation with E-cadherin.

Moreover, the correlation between EMT-related proteins was also determined by Spearman rank test. The results showed that immunohistochemical staining of some EMT-related markers, such as E-cadherin/P-CK, E-cadherin/Slug, and TGF-*β*/Slug, was in significant correlation with each other, which further indicated their roles during the development of EMT in KCOT (Figure 3). Although other tested EMT-related markers were not correlated with each other (data not shown), these results reveal an active statue of EMT in KCOT, in view of the complex processes in EMT activation.

### 3.3. Correlation between Immunohistochemical Staining of EMT-Related Markers and MMP-9 in KCOT

**Figure 4:**
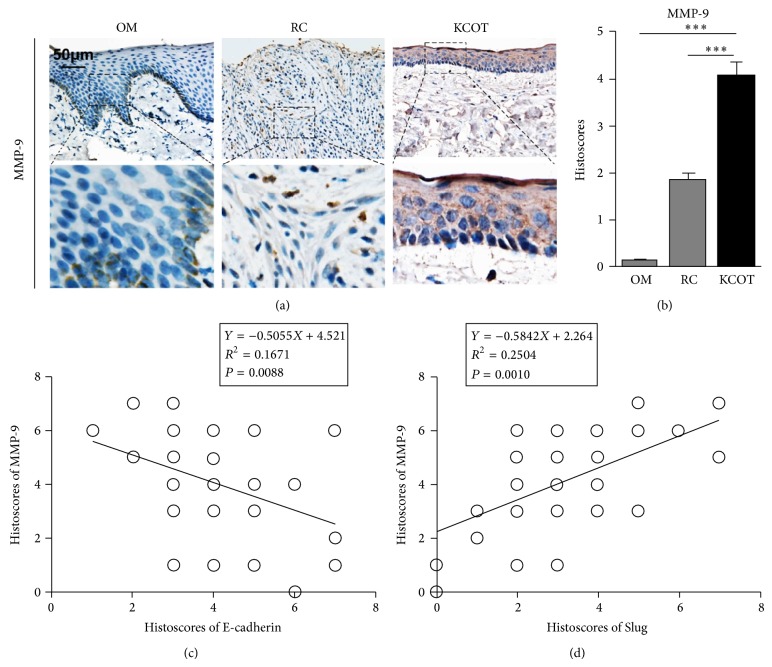
Expression levels of MMP-9 in OM, RC, and KCOT samples, respectively, and correlation analyses for EMT-related markers and MMP-9. (a) The immunohistochemical staining of MMP-9 in KCOT, RC, and OM samples. (b) Immunostaining scores for MMP-9 in KCOT, RC, and OM samples. (c) The expression level of MMP-9 showed a significantly inverse correlation with E-cadherin. (d) The expression level of MMP-9 showed a significantly positive correlation with Slug. Data are presented as means accompanied by SEM. ^***^ *P* < 0.001.

Previous studies have confirmed that EMT could make a great contribution to the invasive property of tumor cells [12, 13]. To further investigate the potential significance of EMT in KCOT, we hypothesized that EMT could also endow more invasive capability to tumor cells in KCOT. Firstly, the expression level of MMP-9, a well-known invasive marker, was detected by using immunohistochemistry. Representative results are demonstrated in Figure 4(a). In line with previous evidences [14], increased expression of MMP-9 in KCOT was found when compared with this in RC and OM (Figure 4(b)). Secondly, the correlation between EMT-related proteins and MMP-9 were determined by using Spearman rank test. Interestingly, the results demonstrated that immunostaining of E-cadherin and Slug was significant correlation with MMP-9, respectively, which indicated the possible role of EMT in the aggressive behavior of KCOT.

### 3.4. Close Relationship between Tested EMT-Related Markers and MMP-9 Reflected by the Hierarchical Clustering Analysis

**Figure 5:**
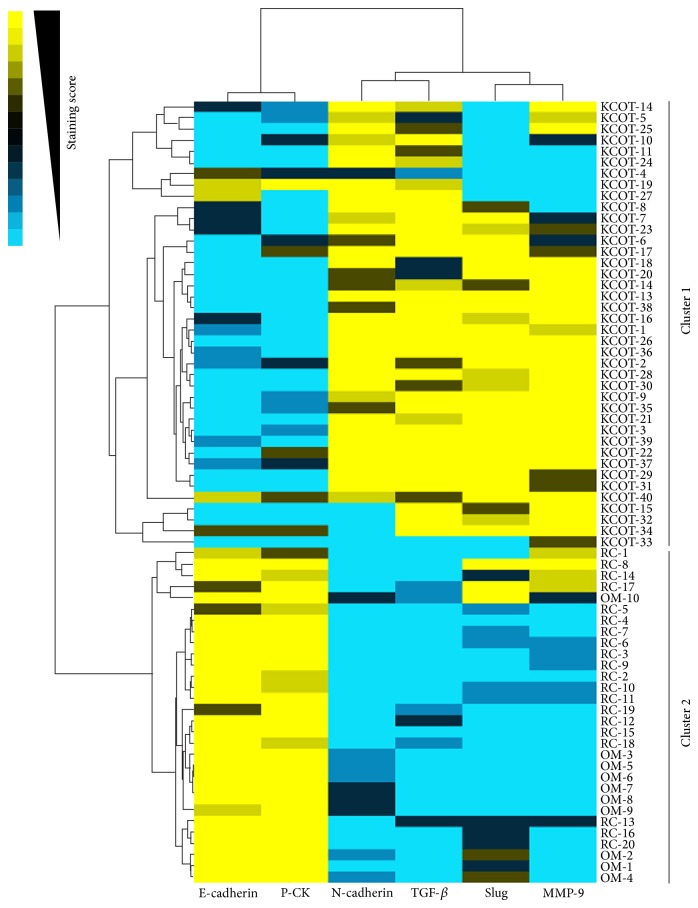
Clustering analysis for tested markers in 40 KCOT, 20 RC, and 10 OM samples. The relationship between indicated markers (top) was visually reflected by the length and subdivision of the branches in the heat-map. In addition, all of the KCOT cases (Cluster 1) and all of the RC and OM cases (Cluster 2) clustered together, revealing the significant difference.

To present our findings more visually, these results were further reflected by the cluster analysis and then visualised by a heat-map. Generally, all the OM and RC samples were strongly positive for E-cadherin and P-CK and nevertheless negative or weakly positive for N-cadherin, TGF-*β*, and Slug as well as MMP-9. By contrast, the expression levels of E-cadherin and P-CK were significantly downregulated, while the expression levels of N-cadherin, TGF-*β*, Slug, and MMP-9 were obviously upregulated in most KCOT samples (Figure 5).

In addition, the correlation between tested markers (top) and samples (left) was showed by the length and subdivision of the branches in the heat-map. As expected, the tested EMT-related markers were closely clustered. Besides, the cluster analysis also demonstrated the closest relationship between E-cadherin and P-CK and Slug and MMP-9, respectively, which was in line with the results from the Spearman rank test. Moreover, all the KCOT samples were clustered together and differed from other clusters of RC and OM samples (Figure 5). Collectively, the cluster analysis declared again the close relationship between the EMT-related markers and their close correlation with locally aggressive behavior of KCOT.

### 3.5. Colocalized Expression of Slug and MMP-9 in KCOT

**Figure 6:**
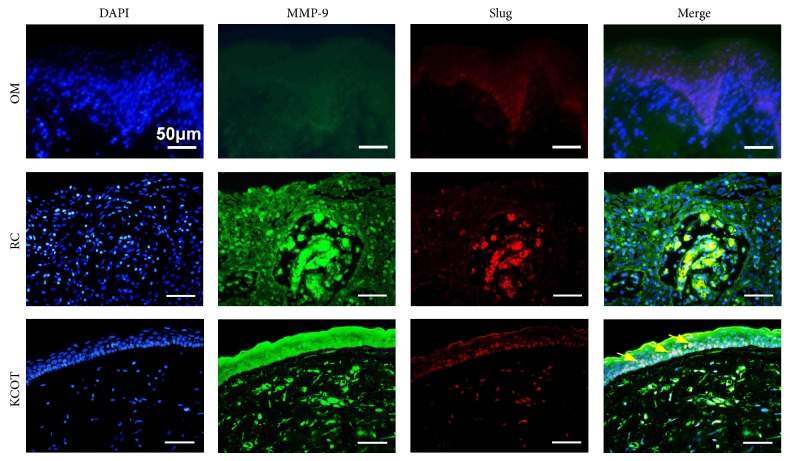
Double-labeling immunofluorescence staining for Slug and MMP-9 in OM, RC, and KCOT tissue samples. The results showed a synchronous expression for Slug and MMP-9 in KCOT as the yellow arrows indicated.

To further explore the possible correlation between EMT and invasive potential, double-labeling immunofluorescence analyses for Slug and MMP-9 were conducted on KCOT clinical samples. As shown in Figure 6, the intense staining of Slug, suggestive of EMT activation, and MMP-9 could be frequently observed in the epithelium of KCOT sections. In addition, synchronous distribution for Slug and MMP-9 signals could be further found in KCOT samples. In contrast, although positive immunofluorescent staining for MMP-9 could be frequently found in the epithelial component of RC samples, colocalizations of Slug with the MMP-9 signals were rarely found in the epithelium of RC and OM samples.

## 4. Discussion

In the present study, we investigated expression levels of EMT-related genes and proteins in KCOT and meanwhile explored the potential role of EMT in the locally aggressive behavior of KCOT.

Distinct from other odontogenic jaw cysts, KCOT is considered to be more locally invasive, rapidly proliferative odontogenic lesions arising from epithelial elements in the jaw. Numerous evidences supporting the more invasive biologic behavior of KCOT were related to the upregulative expression of MMPs [15, 16]. To date, more than 20 MMPs have been identified during the tumor invasive processes, owing to their ability to degrade the extracellular matrix [17]. The expansion of odontogenic tumors occurs through bone destruction. During bone resorption, bone matrix is degraded by enzymes, such as MMP-2 and MMP-9, and the room for neoplasms growth is obtained [18, 19]. In addition, MMP-9 was found to be upregulated in the epithelial component in KCOT and to play a vital role in angiogenesis and tumor invasion as well as in bone resorption in KCOT [14, 15, 20]. Although the strong expression of EGF and ERK-1/2 was thought to have a possible relationship to the overexpression of MMP-9, the exact mechanism behind was largely controversial [21]. In our present studies, we showed that the upregulated expression of MMP-9 was closely related to Slug, a key transcription factor for EMT.

EMT is a process associated with the changes of multiple cell surface proteins, mainly including the downregulation of cytokeratin and E-cadherin. E-cadherin is a specific marker for epithelial cells and the decreased expression of E-cadherin has been regarded as the core event for EMT. Cytokeratin is a primary structural protein of epithelial cells, which is also downregulated in the process of EMT [22, 23]. Notably, it has been indicated that the patterns of cytokeratin are changed in recurrent and syndrome KCOT [24, 25], but the exact mechanism behind this phenomenon is also not clear. Additionally, it is another typical process in EMT that epithelial cells gain the features of mesenchymal cells, especially the upregulation of mesenchymal cell markers N-cadherin [12, 13]. Meanwhile, multiple extracellular signals including TGF-*β*, Notch1, EGF, and hypoxia can initiate the EMT process. Subsequently, significant cross talks among the downstream intracellular transcription factors (e.g., Snail, Slug, and Twist) are activated, which are responsible for dedifferentiation of epithelial cells and gaining mesenchymal cell phenotypes [2, 12]. In our present study, we demonstrated that the expression levels of P-CK and E-cadherin were significantly downregulated, while N-cadherin, TGF-*β*, and Slug were upregulated in KCOT compared with those in RC and OM by using qPCR and immunohistochemistry. Moreover, some tested EMT-related markers showed significant correlation with each other in KCOT. All these findings indicated a possible occurrence of EMT in KCOT.

Recently, increasing evidences including our previous studies asserted that the expression level of MMPs was closely associated with EMT [4, 26, 27]. In various cancer cells, TGF-*β*-induced EMT could upregulate the expression of MMP-2 and MMP-9, the two most critical enzymes, by activating the transcription factor Slug and Snail [28, 29]. Meanwhile, Slug could also cooperate with Snail or other transcription factors to maintain longer-term EMT in cancer cells, by upregulating MMP-9 expression [29]. For the past years, this noted “vicious cycle” theory in cancer was deeply elaborate. In our present experiment, a synchronous distribution for Slug with MMP-9 in KCOT samples was noticed, which implicated that a profound effect of EMT took in the locally invasive processes of KCOT.

## 5. Conclusions

Our present study for the first time demonstrates the activation of EMT and its potential link with aggressiveness observed in KCOT. Specific targeting of the EMT process may further advance the treatment of KCOT. However, further investigation is still needed to deeply confirm the precise mechanism behind correlation between EMT and pathological processes in KCOT.

## Supplementary Material

Summary of clinical features of KCOT and RC patients as well as primer sequences used for real-time quantitative PCR.
