# Supplementary material for: Epithelial-Mesenchymal Transition in Keratocystic Odontogenic Tumor: Possible Role in Locally Aggressive Behavior
Source: Biomed Res Int. 2015 Mar 23;2015:168089. doi: 10.1155/2015/168089 (PMC4386571; doi:10.1155/2015/168089)
Supplement: Supplementary file 1 — Summary of clinical features of KCOT and RC patients as well as primer sequences used for real-time quantitative PCR. [file 168089.f1.pdf]

Supplementary Information:

TABLE S1: Summary of clinical features of keratocystic odontogenic tumor patients.

Patient NO.	Gender	Age (years)	Location	Therapy
1	M	41	Mandible	Curettage
2	F	43	Mandible	Curettage
3	F	36	Mandible	Curettage
4	M	35	Mandible	Curettage
5	M	41	Maxilla	Curettage
6	M	35	Mandible	Curettage
7	F	38	Mandible	Curettage
8	F	27	Mandible	Curettage
9	M	68	Mandible	Curettage
10	M	12	Mandible	Curettage
11	F	28	Mandible	Curettage
12	F	35	Mandible	Curettage
13	F	54	Mandible	Curettage
14	M	71	Mandible	Curettage
15	M	14	Mandible	Curettage
16	M	24	Mandible	Curettage
17	F	34	Mandible	Curettage
18	M	18	Maxilla	Curettage
19	F	44	Mandible	Curettage

**20**	**M**	**34**	**Mandible**	**Curettage**
**21**	**F**	**28**	**Mandible**	**Curettage**
**22**	**F**	**44**	**Mandible**	**Curettage**
**23**	**M**	**21**	**Mandible**	**Curettage**
**24**	**F**	**21**	**Mandible**	**Curettage**
**25**	**F**	**44**	**Mandible**	**Curettage**
**26**	**F**	**21**	**Mandible**	**Curettage**
**27**	**F**	**30**	**Mandible**	**Curettage**
**28**	**M**	**14**	**Mandible**	**Curettage**
**29**	**F**	**38**	**Mandible**	**Curettage**
**30**	**M**	**35**	**Mandible**	**Curettage**
**31**	**M**	**41**	**Mandible**	**Curettage**
**32**	**M**	**71**	**Mandible**	**Curettage**
**33**	**F**	**33**	**Maxilla**	**Curettage**
**34**	**M**	**38**	**Mandible**	**Curettage**
**35**	**F**	**21**	**Mandible**	**Curettage**
**36**	**M**	**18**	**Mandible**	**Curettage**
**37**	**F**	**44**	**Mandible**	**Curettage**
**38**	**M**	**31**	**Mandible**	**Curettage**
**39**	**F**	**34**	**Mandible**	**Curettage**
**40**	**M**	**58**	**Mandible**	**Curettage**

Table S2: Summary of clinical features of radicular cyst patients.

Patient NO.	Gender	Age (years)	Location	Therapy
1	M	71	Mandible	Curettage
2	F	55	Mandible	Curettage
3	M	19	Maxilla	Curettage
4	M	19	Mandible	Curettage
5	M	46	Mandible	Curettage
6	M	13	Mandible	Curettage
7	M	31	Mandible	Curettage
8	F	51	Mandible	Curettage
9	M	24	Mandible	Curettage
10	F	49	Mandible	Curettage
11	M	63	Mandible	Curettage
12	F	59	Maxilla	Curettage
13	F	21	Mandible	Curettage
14	M	45	Mandible	Curettage
15	M	49	Mandible	Curettage
16	F	64	Mandible	Curettage
17	F	17	Mandible	Curettage
18	F	46	Mandible	Curettage
19	M	36	Mandible	Curettage
20	F	32	Mandible	Curettage

TABLE S3: Primer Sequences Used for Real-time Quantitative PCR.

Gene	Forward (5'-3')	Reverse (5'-3')
18s rRNA	CGGCTACCACATCCAAG GAA	GCTGGAATTACCGCGGCT
E-cadherin	GCTGAGCTGGACAGGGA GGA	ATGGGGGCGTTGTCATTCAC
N-cadherin	AGGGCCTTAAAGCTGCTG ACA	TCATAGTCGAAGACTAAAAGGGAGTC ATAT
Vimentin	TCCAGCAGCTTCCTGTAG GT	CCCTCACCTGTGAAGTGGAT
Slug	AGCAGTTGCACTGTGATG CC	ACACAGCAGCCAGATTCCTC
**TGF-β1**	**GGGACTATCCACCTGCA AGA**	**CCTCCTTGGCGTAGTAGTCG**
